# Target product profile: diagnostic test for *Trypanosoma brucei rhodesiense*

**DOI:** 10.2471/BLT.23.290173

**Published:** 2023-06-26

**Authors:** Gerardo Priotto, Jose R Franco, Veerle Lejon, Philippe Büscher, Enock Matovu, Joseph Ndung'u, Sylvain Biéler, Dieudonné Mumba, Nick Van Reet, Paul Verlé, Vincent Jamonneau, Pere P Simarro, Augustin Kadima Ebeja, Dieudonné Sankara, Daniel Argaw Dagne

**Affiliations:** a Department of Control of Neglected Tropical Diseases, World Health Organization, Avenue Appia 20, 1211 Geneva 27, Switzerland.; b Institut de Recherche pour le Développement, CIRAD, University of Montpellier, France.; c Institute of Tropical Medicine, Antwerp, Belgium.; d Animal Resources and Biosecurity, Makerere University, Kampala, Uganda.; e Foundation for Innovative New Diagnostics–Kenya, Nairobi, Kenya.; f Neglected Tropical Diseases Programme, Foundation for Innovative New Diagnostics, Geneva, Switzerland.; g Department of Parasitology, Institut National de Recherche Biomédicale, Kinshasa, Democratic Republic of the Congo.; h Communicable Disease Unit, World Health Organization Regional Office for Africa, Brazzaville, Congo.

## Abstract

Rhodesiense human African trypanosomiasis is a lethal parasitic infection caused by *Trypanosoma brucei rhodesiense* and transmitted by tsetse flies in eastern and southern Africa. It accounts for around 5% of all cases of human African trypanosomiasis. Currently, there is no simple serological test for rhodesiense human African trypanosomiasis and diagnosis relies on microscopic confirmation of trypanosomes in samples of blood or other tissues. The availability of a simple and accurate diagnostic test would aid the control, surveillance and treatment of the disease. A subcommittee of the World Health Organization’s Neglected Tropical Diseases Diagnostics Technical Advisory Group has developed a target product profile for a diagnostic tool to identify *T. b. rhodesiense* infection. The optimum tool would have a sensitivity and specificity above 99% for detecting *T. b. rhodesiense,* but be simple enough for use by minimally trained health-care workers in unsophisticated peripheral health facilities or mobile teams in villages. The test should yield a qualitative result that can be easily observed and can be used to determine treatment. An antigen test would be preferable, with blood collected by finger-prick. Ideally, there should be no need for a cold chain, instrumentation or precision liquid handling. The test should be usable between 10 °C and 40 °C and between 10% and 88% relative humidity. Basic training should take under 2 hours and the test should involve fewer than five steps. The unit cost should be less than 1 United States dollar.

## Résumé

La trypanosomiase humaine africaine à *T. b. rhodesiense* est une infection parasitaire mortelle causée par *Trypanosoma brucei rhodesiense* et transmise par les mouches tsé-tsé en Afrique orientale et australe. Elle représente environ 5% de l’ensemble des cas de trypanosomiase humaine africaine. À l’heure actuelle, il n’existe aucun test sérologique simple pour l’infection à *T. b. rhodesiense* et le diagnostic repose sur la confirmation microscopique de la présence de trypanosomes dans des échantillons de sang ou d’autres tissus. Fournir un test de diagnostic simple et précis favoriserait la lutte, la surveillance et la prise en charge de la maladie. Un sous-comité du Groupe consultatif technique sur les produits de diagnostic des maladies tropicales négligées de l’Organisation mondiale de la Santé a donc élaboré un profil de produit cible pour un outil visant à détecter une infection par *T. b. rhodesiense*. L’outil le plus adapté présenterait un niveau de sensibilité et de spécificité supérieur à 99% pour la détection de *T. b. rhodesiense*, tout en étant à la portée de professionnels de la santé ayant reçu une formation sommaire, tant dans des structures de santé périphériques basiques qu’au sein d’équipes mobiles dans les villages. Cet outil doit fournir un résultat fiable, facile à interpréter, qui peut servir à établir un traitement. Un test antigénique serait préférable, avec prélèvement de l’échantillon sanguin par le biais d’une piqûre au bout du doigt. Idéalement, l’outil ne doit pas être thermosensible, ni nécessiter un équipement spécifique ou une manipulation de liquides délicate. Le test doit pouvoir être utilisé à une température comprise entre 10 °C et 40 °C, ainsi que dans une humidité relative de 10% à 88%. La formation requise pour son utilisation doit durer moins de deux heures et le test doit être effectué en moins de cinq étapes, Enfin, son coût unitaire doit être inférieur à un dollar américain.

## Resumen

La tripanosomiasis humana africana rhodesiense es una infección letal parasitaria causada por el *Trypanosoma brucei rhodesiense*, y es transmitida por la mosca tse-tsé en África oriental y meridional. Representa aproximadamente el 5% de todos los casos de tripanosomiasis humana africana. Actualmente, no existe ninguna prueba serológica simple para la tripanosomiasis humana africana rhodesiense, y el diagnóstico se basa en la confirmación microscópica de tripanosomas existentes en muestras de sangre u otros tejidos. Una prueba diagnóstica sencilla y precisa ayudaría a controlar, vigilar y tratar la enfermedad. Un subcomité del Grupo Asesor Técnico de Diagnóstico de Enfermedades Tropicales Desatendidas de la Organización Mundial de la Salud ha creado un perfil de producto objetivo para una herramienta de diagnóstico que permita identificar la infección *T. b*. *rhodesiense.* La herramienta óptima tendría una sensibilidad y una especificidad superiores al 99% para detectar la *T. b*. *rhodesiense* y, al ser lo suficientemente sencilla, podrían utilizarla trabajadores sanitarios mínimamente formados, en centros sanitarios periféricos no sofisticados, o bien equipos móviles. La prueba debe arrojar un resultado cualitativo de fácil lectura y que pueda utilizarse para determinar el tratamiento. Sería preferible una prueba de antígenos, con sangre extraída mediante punción digital. Idealmente, no debería ser necesaria la cadena de frío, la instrumentación ni la manipulación de líquidos de precisión. La prueba debe poder utilizarse entre 10 °C y 40 °C, con una humedad relativa de entre el 10% y el 88%. La instrucción básica debe llevar menos de 2 horas y la prueba debe incluir menos de cinco pasos. El coste de la unidad debe ser inferior a 1 dólar estadounidense.

## ملخص

داء النوم الأفريقي البشري الروديسي هو عدوى طفيلية قاتلة يسببها داء النوم البروسي الروديسي وتنتقل عن طريق ذبابة تسي تسي في شرق وجنوب أفريقيا. وهي تمثل حوالي 5% من جميع حالات داء النوم الأفريقي البشري. في الوقت الحالي، لا يوجد اختبار مصلي بسيط لداء النوم الأفريقي البشري الروديسي، ويعتمد التشخيص على التأكيد المجهري لوجود الداء في عينات الدم أو الأنسجة الأخرى. إن توفر اختبار تشخيصي بسيط ودقيق من شأنه أن يساعد في السيطرة على المرض، ورصده، وعلاجه. قامت لجنة فرعية من "المجموعة الاستشارية الفنية لتشخيص أمراض المناطق المدارية المهملة" التابعة لمنظمة الصحة العالمية، بتطوير ملف تعريف منتج مستهدف لأداة تشخيص لتحديد الإصابة بعدوى داء النوم البروسي الروديسي. سيكون للأداة المثالية حساسية وخصوصية أعلى من %99 للكشف عن داء النوم البروسي الروديسي، ولكنها تكون بسيطة بما يكفي للاستخدام بواسطة العاملين في مجال الرعاية الصحية المدربين تدريباً محدودًا في المرافق الصحية الثانوية غير المتطورة، أو الفرق المتنقلة في القرى. يجب أن ينتج عن الاختبار نتيجة نوعية يمكن ملاحظتها بسهولة، ويمكن استخدامها لتحديد العلاج. من المفضل إجراء اختبار المستضد، حيث يتم جمع الدم عن طريق وخز الإصبع. وفي الحالة المثالية، يجب ألا تكون هناك حاجة لسلسلة تبريد، أو لأدوات قياس، أو لمعالجة السوائل بدقة. يجب أن يكون الاختبار قابلاً للاستخدام بين درجتي 10 و40 درجة مئوية، وبين %10 و%88 رطوبة نسبية. يجب أن يستغرق التدريب الأساسي أقل من ساعتين، ويجب أن يشتمل الاختبار على أقل من خمس خطوات. يجب أن تكون تكلفة الوحدة أقل من 1 دولار أمريكي.

## 摘要

非洲人类罗得西亚锥虫病是由*布氏罗得西亚锥虫*引起的致命性寄生虫感染,由非洲东部和南部的采采蝇传播。该疾病约占非洲人类锥虫病所有病例的 5%。目前,还没有针对非洲人类罗得西亚锥虫病的简单的血清学检测方法,其诊断依赖于通过显微镜确认血液或其他组织样本中存在锥虫。简单而准确的诊断测试将有助于疾病的控制、监测和治疗。世界卫生组织被忽视热带病诊断技术咨询组的一个小组委员会制定了用于诊断工具的目标产品简介,以识别*布氏罗得西亚锥虫*感染。最适宜的工具在检测*布氏罗得西亚锥虫*病方面应具有 99% 以上的灵敏度和特异性,但使用时也需足够简单,可由受过最低限度培训的卫生保健工作者在简陋的外围卫生设施中或流动小组在村庄中的使用。该测试应产生易于观察并可用于确定治疗方案的定性结果。最好进行抗原检测,通过刺破手指进行采血。理想情况下,不需要冷链、仪器或对液体进行精密处理。该测试应可在温度为 10 °C 至 40 °C 和相对湿度为 10% 至 88% 的的环境中使用。基础培训应在 2 小时内完成,并且测试应少于五个步骤。单位成本应低于 1 美元。

## Резюме

**Родезийский африканский трипаносомоз человека:**

это смертельно опасная паразитарная инфекция, вызываемая *Trypanosoma brucei rhodesiense* и переносимая мухами цеце в Восточной и Южной Африке. На его долю приходится около 5% всех случаев заболевания африканским трипаносомозом человека. В настоящее время не существует простого серологического теста на выявление родезийского африканского трипаносомоза человека. Диагностика основывается на микроскопическом подтверждении наличия трипаносом в образцах крови или других тканей. Наличие простого и точного диагностического теста будет способствовать контролю, наблюдению и лечению этого заболевания. Подкомитет Технической консультативной группы Всемирной организации здравоохранения по вопросам диагностики забытых тропических болезней разработал целевой профиль продукта для диагностического инструмента для выявления инфекции *T. b. rhodesiense*. Оптимальный инструмент должен обладать чувствительностью и специфичностью выше 99% для выявления *T. b. rhodesiense*, но при этом быть достаточно простым для использования минимально обученными медицинскими работниками в неоснащенных периферийных медицинских учреждениях или выездными бригадами в деревнях. Тест должен давать легко наблюдаемый качественный результат, который может использоваться для определения метода лечения. Предпочтительнее использовать тест на антиген, при этом кровь берется из пальца. В идеале не должно быть необходимости в использовании холодовой цепи, контрольно-измерительных приборов или в точной работе с жидкостями. Тест должен допускать выполнение при температуре от 10 до 40°C и относительной влажности от 10 до 88%. Базовая подготовка должна занимать менее 2 часов, а тест должен состоять менее чем из пяти этапов. Стоимость единицы должна составлять менее 1 доллара США.

## Introduction

Human African trypanosomiasis, also known as sleeping sickness, is a vector-borne parasitic disease caused by infection with protozoan parasites belonging to the genus *Trypanosoma*. These parasites are transmitted to humans by the bites of tsetse flies, which are found only in sub-Saharan Africa. The flies acquire their infection from human beings or from animals harbouring human-pathogenic parasites. Human African trypanosomiasis takes two forms, depending on the parasite subspecies involved: gambiense human African trypanosomiasis and rhodesiense human African trypanosomiasis.^1^

Gambiense human African trypanosomiasis is caused by *Trypanosoma brucei gambiense*, which is found in western and central Africa, and between 2011 and 2020 accounted for 95% (32 275 out of 34 096 infections) of reported cases.^2^ The disease has a chronic evolution: a person can be infected for months or even years without major signs or symptoms. When symptoms become evident, the disease is often already at an advanced stage and the central nervous system is affected.^1^

Rhodesiense human African trypanosomiasis is caused by *T. b. rhodesiense*, which is found in eastern and southern Africa, and accounts for 5% (1821/34 096) of reported cases.^2^ Infection results in an acute illness in which signs and symptoms are generally observed after a few weeks. The disease develops rapidly, often provoking multiorgan failure and invading the central nervous system. Epidemic seasonal outbreaks are frequent.^1^

The control of gambiense human African trypanosomiasis is based on screening populations at risk to identify cases and, subsequently, to initiate treatment that will decrease the disease reservoir. In some settings, screening is complemented by targeted insect vector control. Several tools are available or in the pipeline for the screening and diagnosis of gambiense human African trypanosomiasis, but there are no similar tools for rhodesiense human African trypanosomiasis.^1^

At present, there is no simple serological test that can be used to screen for rhodesiense human African trypanosomiasis, and diagnosis relies on the microscopic observation of trypanosomes in blood or other tissues. Samples are analysed either directly as a blood, chancre or lymph node aspirate smear or using concentration methods, such as capillary tube centrifugation or mini anion-exchange centrifugation for blood, or modified single centrifugation for cerebrospinal fluid. However, these methods require the availability of a microscope, centrifuges, an electricity source and trained laboratory technicians. Recently, the progressive introduction of rapid diagnostic tests for malaria has resulted in less equipment being available in peripheral health facilities and a reduced capacity for microscopy examination in rural Africa. Consequently, it is less likely that rhodesiense human African trypanosomiasis will be diagnosed incidentally, which often occurred when microscopy was used to search for malaria parasites.

The availability of a simple test for rhodesiense human African trypanosomiasis would facilitate the control and surveillance of the disease. Treatment could be prescribed more quickly, and the test could help capture additional information on disease transmission, thereby compensating for the ongoing loss of surveillance capacity and possibly even enabling surveillance to exceed previous levels of sensitivity. To achieve this, it is important that testing for rhodesiense human African trypanosomiasis is performed at locations where people seek a malaria diagnosis.

## Method

The World Health Organization’s (WHO) Department of Control of Neglected Tropical Diseases led the development of a target product profile for a diagnostic tool to identify individuals with rhodesiense human African trypanosomiasis that is usable in poorly equipped, peripheral health facilities in areas where there is a risk of disease transmission. During this process, WHO’s standard guidance for target product profile development was followed.^3^ A subgroup on the diagnostic needs of human African trypanosomiasis was established as part of the WHO Neglected Tropical Diseases Diagnostics Technical Advisory Group, which was formed to identify and prioritize the diagnostic needs of neglected tropical diseases. This Advisory Group of independent experts includes leading international scientists and specialists, including from countries where the disease is endemic. Standard WHO declaration of interest procedures were followed for Advisory Group members.^4^ Initially, a landscape analysis of the products currently available and in development was conducted and salient areas of unmet need were identified. In a series of meetings and remote consultations, the human African trypanosomiasis subgroup identified several scenarios in which possible diagnostic tools could help fill the main gaps in disease detection and control, and arranged these scenarios in order of priority. A template for the development of a target product profile for a rhodesiense human African trypanosomiasis test, which included adaptations to the disease context, was agreed. The first draft of the target product profile (rated as priority no. 1) underwent several rounds of review by subgroup members between September 2020 and February 2021. The ensuing version was reviewed by Neglected Tropical Diseases Diagnostics Technical Advisory Group members, and draft version 0.1 was posted on WHO’s website for 28 days on 9 April 2021 for public consultation with a comment form. The final version received executive clearance on 7 June 2021.

Ideally, a diagnostic test for rhodesiense human African trypanosomiasis would be an antigen detection test in a simple format (i.e. a rapid diagnostic test). Alternatively, however, it could be a molecular assay for *T. b. rhodesiense* deoxyribonucleic acid (DNA) or ribonucleic acid (RNA) or a microscopy-free method that can detect the presence of trypanosomes. The test should provide an immediate result that confirms the presence of infection and that could be used for deciding on treatment. If that is not possible, however, an alternative could be a screening test that, if positive, would be followed by confirmatory microscopic examination (bearing in mind that parasitaemia is usually high). Currently, confirmation of infection is mandatory. If, in the future, a safe and short treatment regimen that could be used more widely were developed, it would be desirable to have a screening test that could identify individuals for treatment.

## Target product profile^5^

### Intended use

Ideally, the diagnostic test should be capable of detecting infection by *T. b. rhodesiense*, including the Zambezi and Busoga subtypes. At a minimum, it should be capable of detecting infection by any species of the subgenus *Trypanozoon*, which comprises *T. b. brucei*, *T. b. gambiense*, *T. b. rhodesiense*, *T. evansi* and *T. equiperdum*, all of which are morphologically indistinguishable. A qualitative test that can detect *T. b. rhodesiense* antigen, DNA or RNA or whole parasites would be ideal but one that detected *Trypanozoon* antigen, DNA or RNA or whole parasites would be sufficient. Although a *Trypanozoon* test would suit diagnostic purposes, it would be less accurate for monitoring *T. b. rhodesiense* epidemiology. The early detection of rhodesiense human African trypanosomiasis can be life-saving. The disease (except for the Zambezi subtype) usually evolves rapidly and the generation of specific antibodies takes 1 to 2 weeks. Hence, an antibody detection test would not provide the early diagnosis needed.

The diagnostic test is intended for use in populations at risk of rhodesiense human African trypanosomiasis. Preferably, the test should analyse whole blood or body fluids that can be collected non-invasively (e.g. tears, saliva or urine). Whatever the sampling method, body fluids and tissue specimens should be collected without discomfort to the individual that is disproportionate to the health benefit. Testing will mostly be performed at the point of care, though under certain conditions specimens may occasionally need to be preserved and transported.

Ideally, the test should clearly identify individuals with rhodesiense human African trypanosomiasis who require treatment. Consequently, the test results would provide reliable data for disease surveillance, and a positive test result would trigger both therapeutic and disease control measures. At a minimum, the test should be able to identify individuals with a suspected infection who could be treated after parasitological confirmation or on the basis of heightened suspicion from the clinical picture.

In practice, the test should be capable of being applied by a minimally trained health-care worker at the point of care either in an unsophisticated peripheral health facility or in a mobile team visiting a village, or at a district-level laboratory or a higher-level facility. However, the closer testing is performed to the community at risk, the better.

### Test performance

As rhodesiense human African trypanosomiasis is an acute lethal disease, the test should be highly accurate in detecting *T. b. rhodesiense*. A false-negative result could lead to a lack of treatment for this potentially fatal disease and to underestimates of disease incidence, whereas a false-positive result could lead to unnecessary treatment and overestimates of incidence. In particular, the test should have a high sensitivity, at least equal to the parasitology tests currently used. Ideally, it should have a sensitivity and specificity above 99% for detecting both subtypes of *T. b. rhodesiense*. At a minimum, it should have a sensitivity above 90% and a specificity above 85% for detecting members of the subgenus *Trypanozoon*. If treatment decisions are to be taken on the basis of test results, the desired specificity will depend on the safety of the medicines available: the less safe, the higher the specificity needed. If the test’s specificity is low, however, secondary parasitological confirmation would be essential. Currently, diagnosis and treatment are based on microscopic identification of protozoa of the subgenus *Trypanozoon*. Epidemiological accuracy would be greater with a specific test for *T. b. rhodesiense*.

The analytical sensitivity of the test should ideally be equivalent to 10 or fewer parasites per mL of blood or, at most, 100 parasites per mL. Tests that can detect antigens or nucleic acid sequences may achieve lower detection thresholds than those detecting whole parasites. The repeatability of the results of different tests performed on the same sample by the same reader should have a *Κ*-value above 0.80 (ideally above 0.90). Similarly, the reproducibility of the results of the same test performed on the same sample by different readers should have a *Κ*-value above 0.80 (ideally above 0.90).

Each test kit should include some means of quality control, which will depend on the test’s format. At a minimum, each test should include an assessment of minimal functionality to indicate it is performing properly. Ideally, positive and negative control specimens that could be assessed in parallel with the test specimen should be available at the point of care. Alternatively, these control specimens could be available at a higher-level facility. However, running negative and positive controls for each sample could triple the cost of each test. Providing one set of control specimens per batch or box of tests would be an alternative. In addition, ideally it would be useful if test kits were supplied with a proficiency panel, which is a collection of specimens of known reactivity used to check diagnostic tests against a standard.

### Regulatory requirements

At a minimum, the test’s components should be manufactured in accordance with Current Good Manufacturing Practice (GMP) or ISO 13485:2016. Preferably, manufacturing should be in accordance with CE marking and compliant with the European Union’s Directive 98/79/EC (IVDD 98/79/EC) and ISO 13485:2016 for the quality management system (QMS). In any case, the quality management system should be clearly defined, and the test must be commercially available on the market. There are no particular requirements for promotional or marketing material.

### Health-care system needs

#### Test operation

Ideally, the test should be suitable for use at a temperature between 10 °C and 40 °C and a relative humidity between 10% and 88%. If this is not possible, an operating temperature range of 10 °C to 30 °C and a relative humidity range of 40% to 70% would be acceptable. In some areas where the disease is endemic (e.g. Malawi and Zambia), the ambient temperature may occasionally be low. Preferably, the test should involve fewer than five steps and there should be no need for precision liquid handling. At a minimum, there should be fewer than 10 steps and the use of simple pipette devices only. Ideally, the result should be available in under 20 minutes or, at a maximum, in under 2 hours. The test is intended for professional use only.

#### Instrumentation

The test should not require instrumentation or a cold chain. Less ideally, a cold chain may be needed. Moreover, if instrumentation is required, it should: (i) be portable or hand-held; (ii) weigh 5 kg or less; (iii) be durable, such that it can be easily and safely transported to the field; (iv) be battery-operated and also able to run on standard mains electricity; (v) be able to function without the need for running water; (vi) be resistant to shock and vibration; and (vii) have a life span of at least 5 years with minimal and easy-to-perform maintenance.

#### Data recording and transmission

The test should be simple to operate manually and yield a qualitative result that can be observed visually or is displayed on a portable device. Ideally, the result should be stable for at least 30 minutes. At a minimum, it should be stable for at least 15 minutes. The specimen identifier could be written in a logbook or entered into a computer or smartphone. Neither the data display nor data entry should require the availability of a digital interface or communications connectivity (i.e. internet or phone). In particular, treatment decisions should not depend on connectivity.

Ideally, test results should be capable of being integrated into national data and reporting systems and of being easily stored for retrospective interpretation (e.g. as electronic results, optical density or intensity measurements, or electronic images or video). The data should be exportable to any database, if necessary, though the amount of data storage needed will depend on the program used. Ideally, the data generated should be capable of being automatically integrated into a database on a server without the need for additional equipment. At a minimum, test results could be entered manually into a computer database and transmitted manually. Data transmission should be flexible, such that, depending on connectivity, data could be sent by e-mail, short message service (SMS) or phone.

#### Test stability and handling

All tests should be packaged individually and accompanied by any accessories required for sample collection and processing, as well as by operating instructions. Ideally, tests should be stable at a temperature between 5 °C and 45 ᵒC and a relative humidity between 40% and 88% for at least 24 months and transportation should not require a cold chain. At a minimum, tests should be stable at a temperature between 4 °C and 8 ᵒC and a relative humidity between 40% and 88% for at least 12 months. In use, tests should ideally be stable for at least 2 hours after the test pouch is opened or, at a minimum, for 30 minutes after opening. Ideally, reagents should be ready to use or require a maximum of two additional steps to be ready to use; at a minimum, they should be ready to use within 15 minutes and require no more than five additional steps. Stability estimates should take into account the time needed for transport from the manufacturer, passage through customs and local distribution.

#### Sample handling

The sample volume will depend on the type of specimen. Ideally, blood would be collected by finger-prick, with a maximum volume of 0.02 mL. However, a capillary tube could easily draw 0.05 mL of blood from a finger-prick. Alternatively, blood, serum or plasma collection may require a maximum volume of 5 mL. The volume is less important for non-invasive sampling. Additional specimen material could be collected at the same time for repeat testing if needed.

Ideally, no special collecting device should be required and there should be no specimen processing. If a collecting device is needed, it should be provided with the kit and specimen processing should be minimal. It is important to note that special collecting devices are not routinely available in peripheral health centres in areas where human African trypanosomiasis is endemic. Occasionally, specimens could be preserved and transported under certain circumstances.

Standard biosafety precautions for handling potentially infectious materials should be taken. Waste should be disposed of in a biosafety bin according to standard guidelines, and sharps containers should be available for the disposal of sharp objects, such as lancets and capillary tubes. Excess specimens and consumables used in the test process should be disposed of using an appropriate method, for example, in a latrine or by incineration. Standard operating procedures should be provided.

#### Training and support

Ideally, the basic training required to use the test should take less than 2 hours or, at a maximum, 1 day. Manufacturers should replace non-functioning tests or instruments and guarantee the availability of tests for at least 7 years after marketing (5 years at a minimum). External support should be available, with a response time of 1 day ideally (1 week at a maximum).

### Sustainability and cost

Tests should continue to be produced even in the absence of a profitable market. The quantity of rhodesiense human African trypanosomiasis tests needed will be smaller than that used for gambiense human African trypanosomiasis, which will probably increase the production cost per unit. As the testing and treatment of human African trypanosomiasis is a non-profit endeavour, funding has to be sustainable and an innovative model for test production and access is vital. Donors could ensure affordability. Hence, advocacy will be essential.

The cost of each test, excluding sample collection costs, should ideally be 1 United States dollar (US$) or less. The maximum cost is US$ 20, which would enable molecular tests to be considered. This amount does not include the cost of hardware, material shipment, sample collection or salaries.

## Conclusions

The human African trypanosomiasis subcommittee of WHO’s Neglected Tropical Diseases Diagnostics Technical Advisory Group has developed a target product profile for a diagnostic tool to identify the presence of *T. b. rhodesiense* infection. The availability of a simple and highly accurate diagnostic test that can be used in remote locations would enable treatment to be prescribed more quickly and, consequently, more lives to be saved. In addition, the test would also help capture more information on disease transmission, thereby compensating for the loss of surveillance capacity that has occurred in rural Africa as rapid diagnostic tests for malaria have replaced microscopy examination.
